# Shaping the Sensory–Motor Network by Short-Term Unresolvable Sensory–Motor Mismatch

**DOI:** 10.3389/fneur.2021.793662

**Published:** 2022-01-12

**Authors:** Carsten M. Klingner, Fabian Kattlun, Lena Krolopp, Elisabeth Jochmann, Gerd F. Volk, Stefan Brodoehl, Orlando Guntinas-Lichius, Otto W. Witte, Christian Dobel

**Affiliations:** ^1^Hans Berger Department of Neurology, Jena University Hospital, Jena, Germany; ^2^Biomagnetic Center, Jena University Hospital, Jena, Germany; ^3^Clinic for Otorhinolaryngology, Jena University Hospital, Jena, Germany

**Keywords:** fMRI, facial palsy, plasticity, motor adaptation, sensory–motor mismatch

## Abstract

Learning from errors as the main mechanism for motor adaptation has two fundamental prerequisites: a mismatch between the intended and performed movement and the ability to adapt motor actions. Many neurological patients are limited in their ability to transfer an altered motor representation into motor action due to a compromised motor pathway. Studies that have investigated the effects of a sustained and unresolvable mismatch over multiple days found changes in brain processing that seem to optimize the potential for motor learning (increased drive for motor adaptation and a weakening of the current implementation of motor programs). However, it remains unclear whether the observed effects can be induced experimentally and more important after shorter periods. Here, we used task-based and resting-state fMRI to investigate whether the known pattern of cortical adaptations due to a sustained mismatch can be induced experimentally by a short (20 min), but unresolvable, sensory–motor mismatch by impaired facial movements in healthy participants by transient facial tapping. Similar to long-term mismatch, we found plastic changes in a network that includes the striatal, cerebellar and somatosensory brain areas. However, in contrast to long-term mismatch, we did not find the involvement of the cerebral motor cortex. The lack of the involvement of the motor cortex can be interpreted both as an effect of time and also as an effect of the lack of a reduction in the motor error. The similar effects of long-term and short-term mismatch on other parts of the sensory–motor network suggest that the brain-state caused by long-term mismatch can be (at least partly) induced by short-term mismatch. Further studies should investigate whether short-term mismatch interventions can be used as therapeutic strategy to induce an altered brain-state that increase the potential for motor learning.

## Introduction

Many neurological patients suffer from motor impairments. These motor impairments cause a discrepancy (or mismatch error) between the desired movement and the executed movement. Often, these patients are unable to reduce this mismatch error by modifying their motor behavior (e.g., due to lesions of the spinal tract or peripheral nerve palsy). In these cases, learning from errors as the main mechanism for motor adaptation becomes ineffective. However, despite this behavioral inefficiency, the sustained mismatch remains a strong driver of motor adaptation, causing distinct changes in the processing of the desired sensory and motor information (1–3). Facial nerve palsy is a particularly well-suited example to study such a mismatch effect because the patient is unable to reduce the mismatch error (attempts to adapt the cerebral motor plan are ineffective due to the block of motor information in the peripheral facial nerve). Previous studies on this disease have suggested that the sensory–motor mismatch (without any reduction in the mismatch error) elicits an increased drive for motor adaptation and a weakening of the current implementation of motor programs (4–7). From a theoretical perspective, it is reasonable to suggest that the mismatch, *per se*, drives brain changes, establishing optimized conditions for motor learning.

To further investigate such a mechanism, we need to know the time course of the induced changes in cerebral processing after the induction of a mismatch. Recent studies have described this effect after several days of persistent facial nerve palsy in multiple brain areas (e.g., the primary and secondary motor and somatosensory cortex, the supplementary motor area (SMA), the putamen, the caudate nucleus and the cerebellum) (5, 6, 8). However, for therapeutic use, much shorter periods of induced mismatch are desirable. The short-term effects of an unresolvable mismatch on the cerebral processing of information are currently not known. By the term “unresolvable mismatch” we refer here to a condition in which it is not possible through learning/adaptation to perform the target movement correctly. There remains a divergence between the intended movement and the movement executed.

In the current study, we aim to investigate whether the general pattern of cerebral adaptation that was observed after persistent mismatch across multiple days in observational cohort studies (4–7) can be induced experimentally by applying a short-term (20 min) mismatch to facial movements that cannot be resolved by updating the motor program. Based on the results of the available studies in patients with subacute facial palsy, we hypothesize that short-term mismatch similarly induces increased connectivity between the cerebellum and the basal ganglia, as well as a decreased functional connectivity in the sensory cortex and motor cortex.

We investigated these hypotheses in the present study by employing functional magnetic resonance imaging (fMRI) in healthy participants before and after the experimental induction of a short-term sensory–motor mismatch.

## Materials and Methods

### Participants

Thirty-eight volunteers without any history of neurological, otolaryngological, or psychiatric diseases participated in this study. Three participants had to be excluded because of incomplete data, and another subject exhibited movement artifacts during MRI acquisition larger than 3 mm and was therefore excluded from further analyses, resulting in a final group size of 34 participants (age 23.7 ± 4.5 years ranging from 20 to 33, 16 male, 18 female). Handedness was assessed by the Edinburgh Inventory (9), which ranges from −100 for strong left handedness to +100 for strong right handedness. Only right-handed (> +79) participants were included. The study was approved by the local ethics committee (5519-04/18), and all patients gave their written informed consent according to the Declaration of Helsinki.

### Experimental Induction of Impaired Facial Function

**Figure 1:**
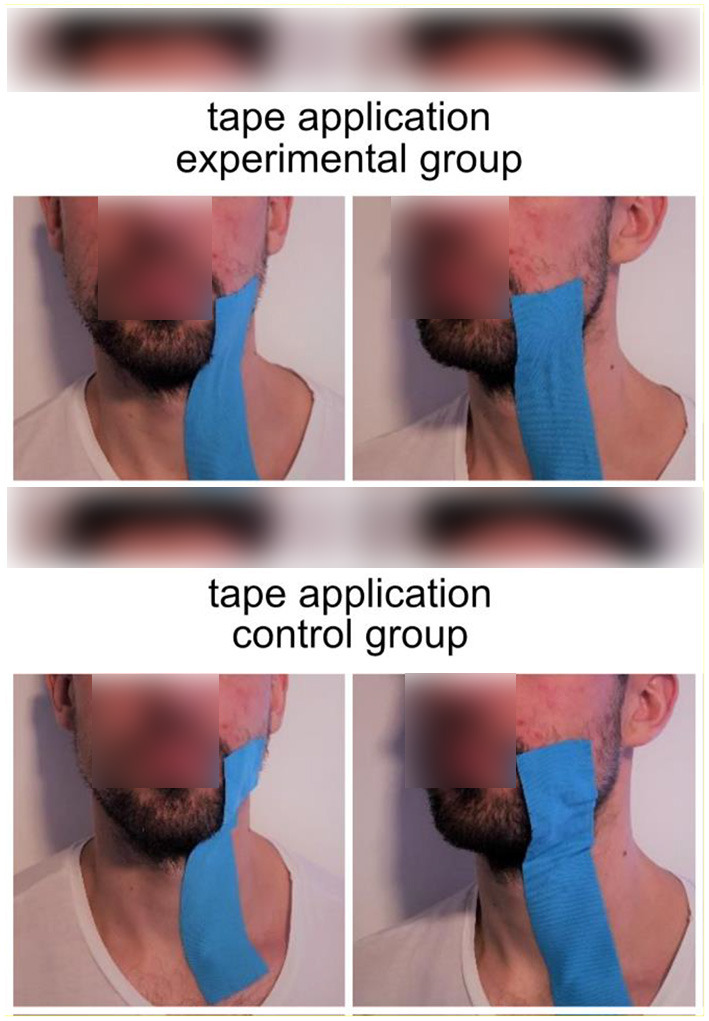
Tape application in the experimental group (upper part) and the control group (lower part of the image). Blue kinesiology tape was applied between the left corner of the mouth and the clavicle. In the experimental group, the tape was shortened by 40%, resulting in a downward pull on tissue on the left side of the face. Photos were restaged after the completion of the experiment.

The experiment aimed to compare the effect of impaired facial movements against a control/sham group. To impair facial movements in healthy participants, we used kinesiology tape (Kinesio Tape, Nasara, Germany) with a width of 5 cm (see Figure 1). This tape created a downward pull of tissue on the left side of the face that impaired the participants' ability to move the left side of their face. The resulting impairment in the ability to move the face also impairs the ability to speak clearly, resulting in slight dysarthria. In the control/sham group, we also applied the tape but without any downward pull (sham taping). Participants were randomly assigned to the experimental or the control group. The type of tape application was the only difference between the control and experimental group protocols.

The taping procedure was conducted as follows. To create the same downward pull for all participants, the tape length was specified individually by measuring the distance between the left corner of the mouth and the clavicle. The tape was applied between the left corner of the mouth (zygomaticus major) and the clavicle in all participants. However, in the experimental group, the tape was shortened by 40%. This created an involuntary downward pull of the left side of the face in the experimental group (see Figure 1).

### Clinical Assessment of Facial Function

After tape application, standardized photographs of different facial expressions were taken for facial nerve grading according to the Sunnybrook Facial Grading System (10). These pictures were later evaluated by reviewers who were blinded to the study and the groups of participants. The Sunnybrook Facial Grading System includes an evaluation of the facial symmetry at rest, the facial symmetry of voluntary movement and the degree of involuntary muscle contractions associated with each expression (synkinesis). The composite score of these three subscores was estimated to range from 0 (complete facial palsy) to 100 (no visible sign of a facial palsy).

### General Experimental Procedure

**Figure 2:**
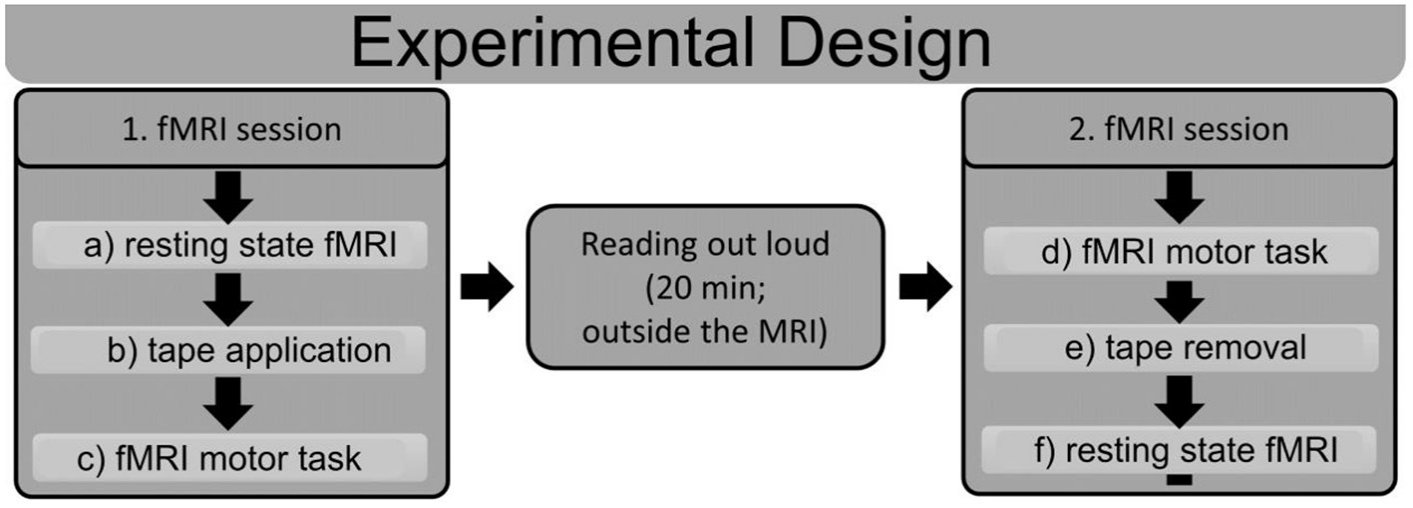
Schematic outline of the experimental design. Subjects underwent two fMRI sessions with a reading task in between. The first fMRI session started with a resting state fMRI **(a)**, after which the tape was applied in the MRI machine **(b)**. Subsequently, subjects performed a motor task in the MRI machine **(c)**. After the first MRI session, subjects read out loud for 20 min outside of the MRI machine. The second fMRI session started with the same motor task as that in the first session **(d)**; afterwards, the tape was removed **(e)**, and another resting state fMRI scan **(f)** was performed. ROIs were derived from the fMRI motor task **(c,d)**, and connectivity analyses were performed for the resting state fMRIs **(a,f)**.

The experiment consisted of two MRI sequences with a behavioral task in between. Figure 2 shows a schematic outline of the experimental procedure. The first MRI session started with a resting-state measure. Upon completion, we applied the tape while participants were in the MRI machine, and the participants subsequently performed a facial motor task (see below) during an fMRI scan that was used as a functional localizer in further analyses. After the motor task, the participants were removed from the MRI machine. Next, they were required to read a book (“Harry Potter” by Joanne K. Rowling) out aloud for 20 min. The reading task was chosen in such a way that participants were forced to use their facial muscles, which produced a discrepancy between intended and executed movement in the tape group, whereas no such discrepancy occurred in the shame group.

After this, participants were again tested in the MRI machine. In this second session, the fMRI motor task was performed first, and then, the tape was removed, and the second resting state measurement was performed. The rationale for the inverse sequence in the second MRI session was to access the motor task consistently with impaired facial function in both measurements, as well as acquiring both resting state measurements without the tape.

### Experimental Design of the MRI Facial Motor Task

Participants were instructed to raise the left corner of the mouth and then relax their facial muscles to resume the starting position. The motor task was performed at a frequency of 1 Hz for 24 s, followed by a 24-s rest (7 blocks). The pace was set visually. The participants saw the words “Pull up the left corner of your mouth” (in German “Ziehen sie den linken Mundwinkel nach oben”) and below this text the word “now.” The Word “now” (in german “Jetzt”) became visible and disappeared again with the frequency with which the subjects should move the corner of their mouth upwards. This movement paradigm was practiced with the subjects before starting the study.

### MRI Recordings

All examinations were performed on the same 3.0 Tesla MR scanner (Trio, Siemens, Erlangen, Germany) to obtain echo-planar T2^*^-weighted image volumes (EPI) and transaxial T1-weighted structural images. Functional resting state data were acquired in two EPI sessions of 250 volumes. The patient was instructed to lie down with his or her eyes closed, to think of nothing in particular, and not fall asleep. The first 3 volumes were subsequently discarded due to equilibration effects. A functional image volume was composed of 100 transaxial slices, including the whole cerebrum and cerebellum (voxel size = 1.4 mm × 1.4 mm × 1.4 mm, repetition time = 1.95 s, TE 33.6 ms). Task-related fMRI sessions were performed after the resting state scan, during which 710 images (56 transaxial slices each, voxel size = 2.5 mm × 2.5 mm × 2.5 mm, repetition time = 0.484 s, TE 30 ms) were acquired. The first 3 volumes were subsequently discarded due to equilibration effects. After functional measurement, high-resolution T1-weighted structural images (voxel size = 1 mm × 1 mm × 1 mm) were acquired.

### Pre-processing of Functional Data (Resting State and Facial Motor Task)

Preprocessing was performed on a workstation using MATLAB (Mathworks, Natick, MA) and SPM12 software (Wellcome Department of Cognitive Neurology, London UK; http://www.fil.ion.ucl.ac.uk/spm). For each subject, all images were realigned to the first volume using a six-parameter rigid-body transformation that corrected for motion artifacts. The images were coregistered with the subject's corresponding anatomical (T1-weighted) images, resliced to correct for acquisition delays, normalized to the Montreal Neurological Institute (MNI) standard brain (11) to report MNI coordinates, and smoothed using a 6-mm full-width-at-half-maximum Gaussian kernel.

### fMRI Analysis of the Functional Motor Task

Multiple regression analysis using a general linear model was performed to obtain statistical parametric maps calculated for the motor task. Functional MRI signal time courses were high-pass filtered (128 s) and modeled as an experimental stimulus onset function, convolved with the canonical hemodynamic response function (low-pass filter). Individual results were projected onto the coregistered individual high-resolution T1-weighted 3-D data set. The anatomical localization of activations was analyzed with reference to a standard stereotaxic atlas and by visual inspection of the individual T1-weighted structural data. A FWE-corrected *p*-value (*p* < 0.05) served as the threshold for the resulting statistical maps.

### Connectivity Analysis of Resting State Data

Functional connectivity is a measurement of the temporal correlations of low-frequency (<0.1 Hz) blood oxygenation level dependent (BOLD) fMRI signal fluctuations between distinct brain areas (12, 13). Most studies examine functional connectivity in the resting state, in which these BOLD fluctuations are presumed to relate to “spontaneous” neural activity and reflect information transfer and collaboration between brain areas (12, 14). Changes in functional connectivity within the facial motor network were investigated in the resting state. To identify relevant areas of the facial motor network, we used activation maps obtained from the motor task (Figures 2c,d). Since there were two fMRI sessions for each subject, we concatenated both sessions before the group analysis. From each activated region, we selected all voxels that demonstrated significant activity in the facial motor task (*p* < 0.05; FWE corrected), and these were further used as regions of interest (ROIs). The resting state data (Figures 2a,d) from these identified ROIs were extracted, and cluster-specific time series were estimated by averaging the time series of all voxels within a cluster. Several sources of variance, including (1) six parameters obtained by rigid body correction of head motion, (2) the signal from a ventricular ROI and (3) the signal from a region centered in the white matter, were then removed from the data by linear regression. All signal intensity time courses were bandpass filtered (0.01 < *f* < 0.1) to reduce the effect of low-frequency drift and high-frequency noise. Pearson's correlation coefficient was computed between all ROIs for each subject. Then, we estimated the network-specific connectivity.

All regions of interest were assigned to one of four functional networks according to the hypothesis of our study (sensory–motor cortical network; basal ganglia; cerebellum). To access the WITHIN network connectivity, we estimated the connectivity between each combination of regions that belong to this network. To access the BETWEEN network connectivity, we estimated the connectivity between each combination of regions that exists between two networks. This analysis was performed separately for the first and second resting state measurements. We then estimated the difference between the connectivity values by subtracting the individual *r*-value for a specific connection of the first measurement from the *r*-value of the second measurement. The resulting *r*-value was then transformed to a *z*-score by Fisher's *r*- to *z*-transformation. All *z*-values of all connections of interest (e.g., all connections between the cerebellum and the basal ganglia subregions) were then averaged for each participant. The resulting connectivity values (within subject difference) were then compared between the experimental group and the control group.

First, we performed an Analysis of Variance on the connectivity *z*-values, with the respective within (different networks) and between-subject (groups) factors. The *z*-scores were further analyzed according to our hypotheses by two-sample two-sided *t*-tests to determine whether the two groups (experimental vs. control) showed significantly different functional connectivity. To account for the number of hypotheses and tests, the alpha value (*p* < 0.05) was corrected for these multiple comparisons (12 tests) using the Šidák correction. Accordingly, findings were considered significant at *p* < 0.0043.

## Results

### Clinical Assessment of Facial Function

After tape application to the left side of the face, all subjects in the experimental group showed a unilateral decline in facial function with a mean Sunnybrook composite score of 80.9 ± 7.6, while the control group showed a Sunnybrook composite score of 94.4 ± 5.1. The composite score was significantly different between groups (*p* < 0.001, *t* = −5.9, df = 28). All participants in the experimental group, but none in the control group, clearly showed slurred speech.

### fMRI of the Facial Motor Task

**Figure 3:**
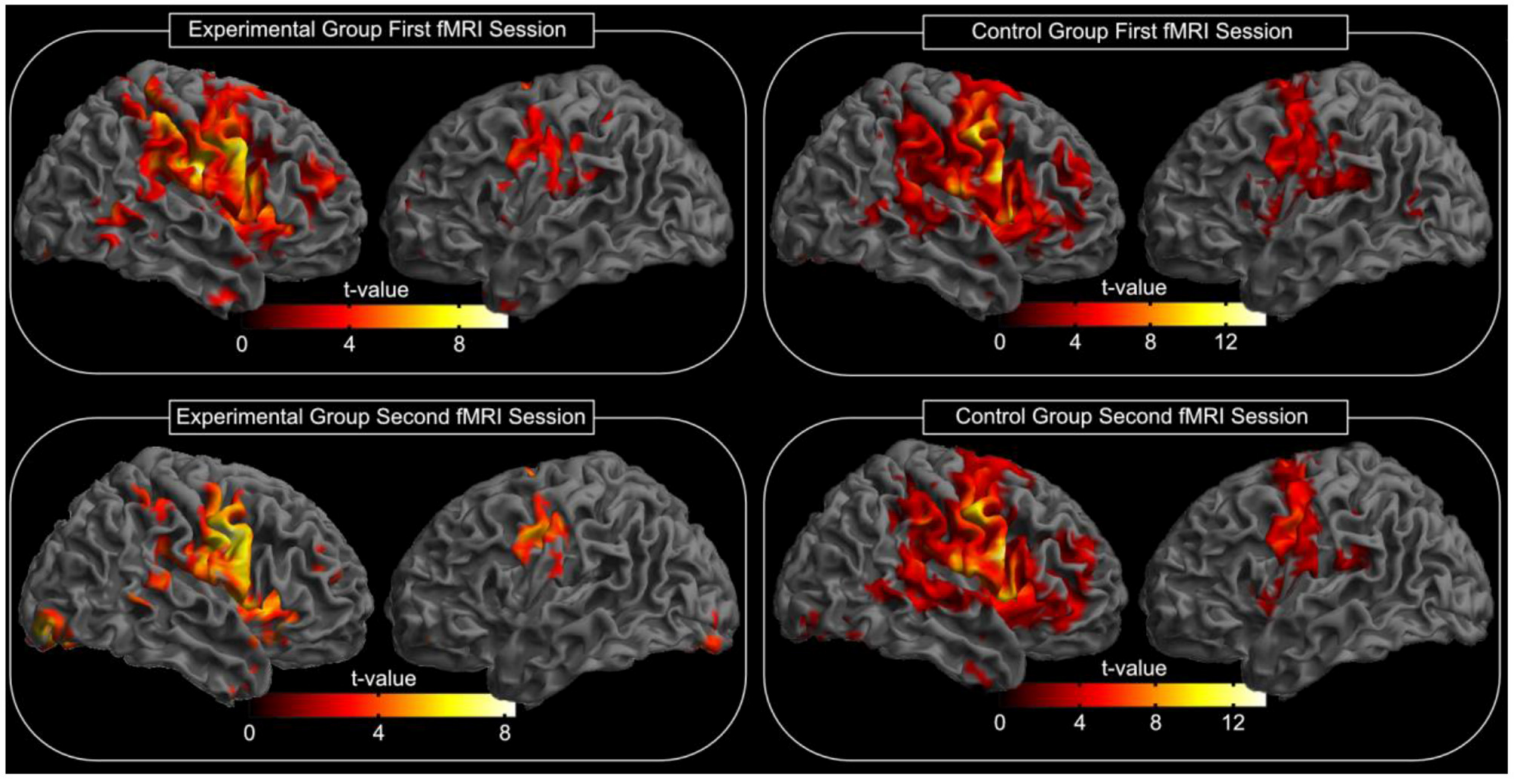
Random effects group analysis of the facial motor task. Activations (*p* < 0.05, FWE corrected) in response to the blocked (24 s) movement of the left side of the face are shown superimposed on a template cortex. The left part of the image shows the experimental group, while the right part shows the results of the control group. The upper part of the image shows the results of the first measurement; the lower part shows the results of the second measurement (after reading; see also Table 1 for spatial location and *t*-values). The test for differences between the first and the second fMRI session for each group did not reveal significant changes in the activation pattern (*p* < 0.05, FWE corrected).

After tape application to the left side of the face, all participants performed a facial motor task with the left side of the face taped during the acquisition of an fMRI scan. This task evoked highly significant activations in each subject and in the random effect group analysis (*p* < 0.05, FWE corrected, Figure 3). At this significance level, the bilateral MI, bilateral LPMCv (MII), bilateral SMA, bilateral SI, bilateral SII, bilateral putamen, contralateral caudate nucleus, contralateral thalamus and the ipsilateral cerebellum were found to be activated (Figure 3). The *t*-values and MNI coordinates for the random effect analyses of the motor task are summarized in Table 1. The spatial locations of activated clusters were further used in the following connectivity analysis.

**Table 1:** MNI coordinates of fMRI activation maxima with the corresponding *t*-value for the motor task.

	**Before**				**After**			
	**x**	**y**	**z**	***t*-value**	**x**	**y**	**z**	***t*-value**
MI c	53	3	40.5	6.1	55	−4.5	35	8.0
MI i	−59.5	−2	37.5	5.1	−57	−4.5	40	5.7
MII c	55.5	3.5	24.5	6.8	58	3	22.5	6.6
MII i	−54.5	5.5	25	4.4				
SMA c	3.5	−2	67.5	4.7	3	−4.5	65	4.7
SMA i	−2	−5	70	4.5	−2	−4.5	65	4.6
SI c	55.5	−14.5	32.5	10.4	48	−14.5	35	5.6
SI i	−49.5	−17.0	32.5	6.1	−47	−14.5	32.5	6.6
SII c	53	−27	22.5	5.1	49	−22	28	3.7
SII i	−59.5	−25.5	20.5	4.9				
Ncl. Caud. c	25.5	15.5	7.5	7.0	15.5	3	20	3.9
Putamen a c	27.5	12	10.5	4.9	28	10.5	7.5	4.4
Putamen p i	−19.5	−2	7.5	3.8				
Putamen p c	33	−1	10.5	5.4	34	−7	10	5.1
Thalamus c	20.5	−17	9	3.8	20.5	−17	10	6.2
Cereb Lob V i	20.5	−62	−17	4.5	20.5	−60.5	−18.5	6.6
Cereb Lob VI i	−29.5	−59.5	−25	5.0	−29.5	−54.5	−32.5	6.6
Cereb Lob VIIIa i	−9.5	−67	−47.5	5.8	−10	−65	−50	6.9

*MI, primary motor area; MII, secondary motor area / ventral lateral premotor cortex; SI, primary somatosensory cortex; SII, secondary somatosensory cortex; SMA, supplementary motor area; Cereb, cerebellum; Put, putamen; N Caud, caudate nucleus; Thal, thalamus; c, contralateral; I, ipsilateral; p, posterior; a, anterior*.

The test for differences between the first and the second fMRI session for each group did not reveal significant changes in the activation pattern (corrected for multiple comparisons using FWE, *p* = 0.05). Additionally, we compared whether the difference between both fMRI sessions differs between the experimental group and the control group. However, no significant changes in the activation pattern were found (corrected for multiple comparisons using FWE, *p* = 0.05).

### Functional Connectivity

The functional connectivity in the resting state was estimated between different cortical and subcortical brain regions. The spatial locations of the included brain areas were determined by the activity map of the facial motor task. The correct assignment of activations to cortical regions of interest was verified using the AAL atlas and the anatomy toolbox. We found three activated subregions within the cerebellum, three activated subregions within the basal ganglia, one active cluster within the thalamus, two active subregions within the cortical somatosensory cortex and three active subregions within the cortical motor cortex (Table 1). The difference in the functional connectivity between the two measurements (before and after reading) was estimated for all participants and compared between the two groups (taping and control).

We further tested for group differences in the functional connectivity within each of our 4 main subnetworks by using the Student's *t*-test (motor cortex, sensory cortex, basal ganglia and cerebellum). We found significantly increased connectivity within the basal ganglia (*t* = 3.8; df = 33; *p* < 0.001; *r* = 0.53) and within the cerebellum (*t* = 3.1; df = 32; *p* = 0.003; *r* = 0.48), while no altered connectivity was found in the cortical somatosensory network (*t* = 0.5; df = 32; *p* = 0.61; *r* = 0.09) or the motor network (*t* = 0.8; df = 32; *p* = 0.43; *r* = 0.14).

**Figure 4:**
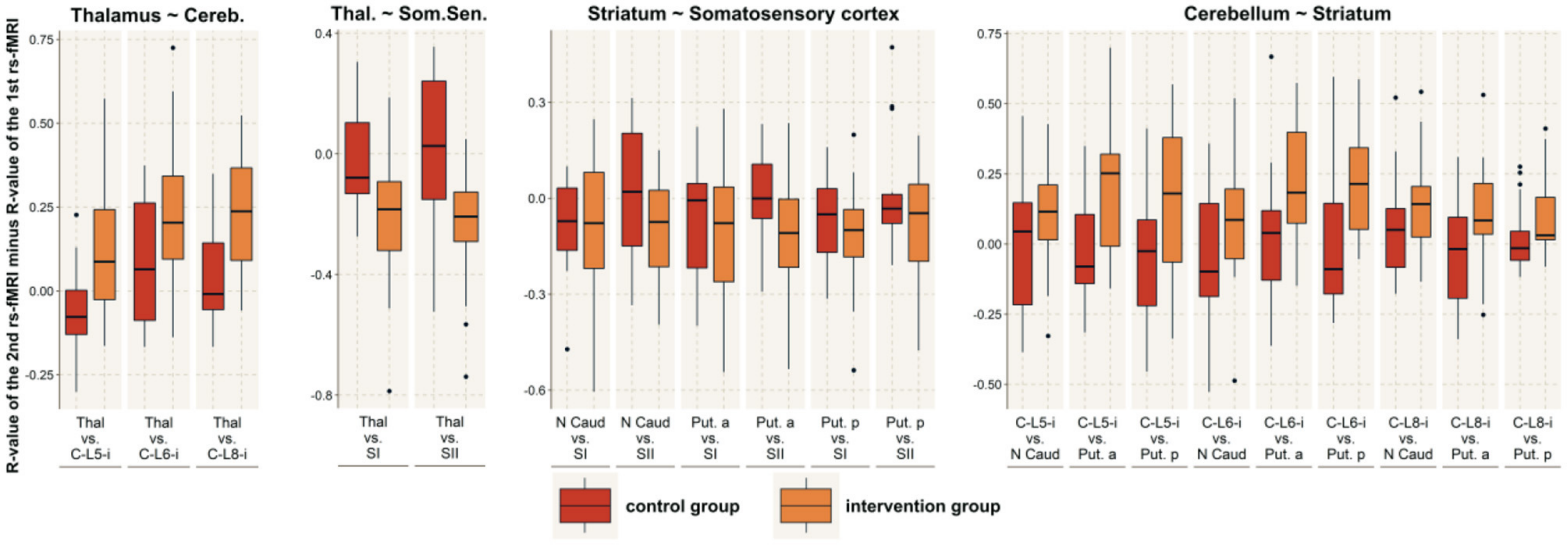
Between-network connectivity. The figure shows the R-values of the Pearson correlation of the 2nd rs-fMRI minus the R-value of the 1st rs-fMRI for those brain networks that demonstrated significantly altered functional connectivity with another network. For those connected networks, the image shows all the connections between brain areas that are part of the respective network. The red boxes indicate the control group, while the orange boxes indicate the intervention group. MI, primary motor area; MII, secondary motor area / lateral premotor cortex; SI, primary somatosensory cortex; SII, secondary somatosensory cortex; C, cerebellum; Put, putamen; N Caud, caudate nucleus; Thal, thalamus; p, posterior; a, anterior.

**Figure 5:**
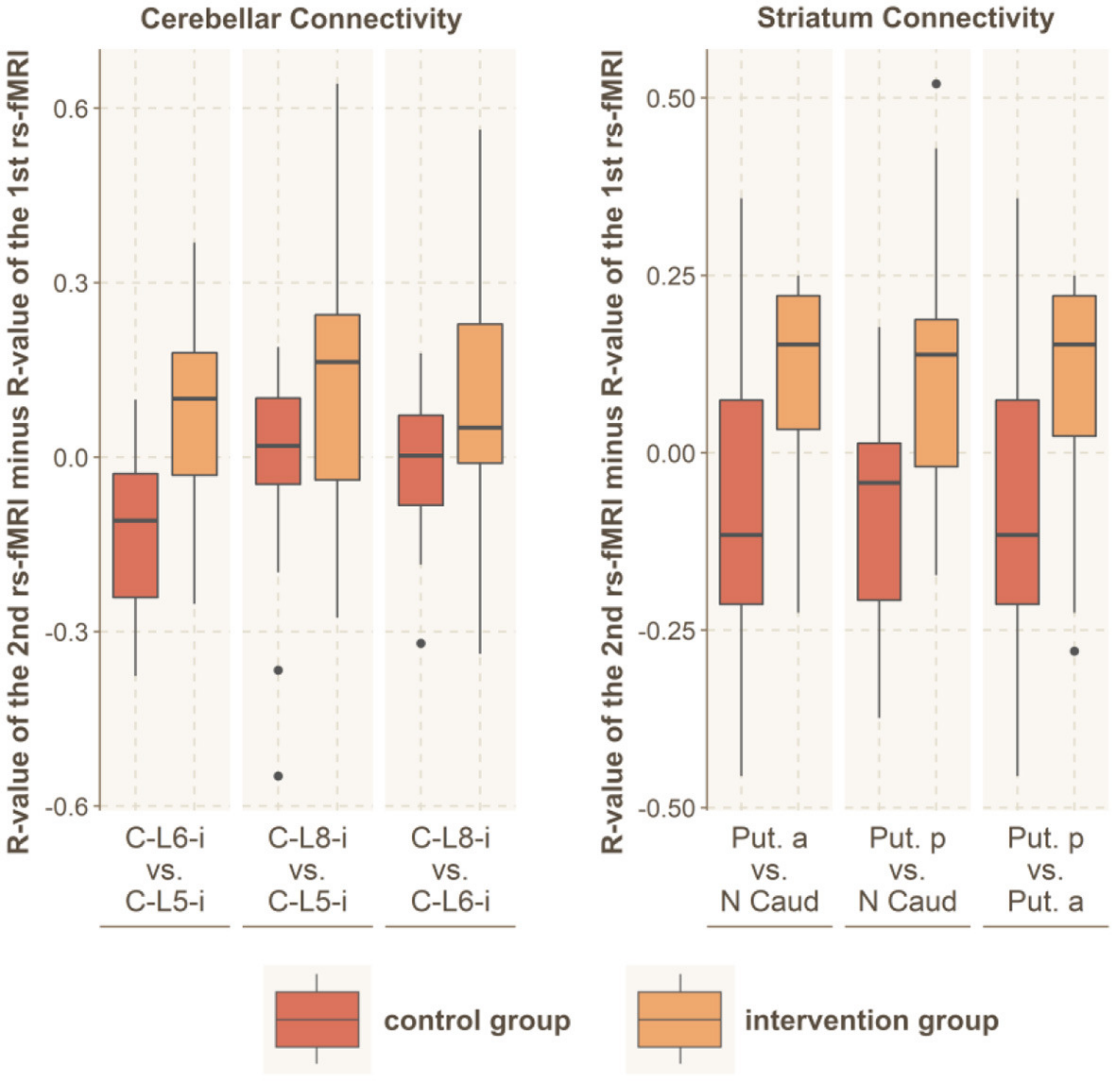
Within-network connectivity. Significantly altered within-network functional connectivity was found for the striatum and the cerebellar network. The figure shows the R-values of the Pearson correlation of the 2nd rs-fMRI minus R-value of the 1st rs-fMRI for all connections within these brain networks. The red boxes indicate the control group, while the orange boxes indicate the intervention group. C, cerebellum; L, lobe; Put, putamen; N Caud, caudate nucleus.

Concerning changes between active brain networks, we found significantly increased connectivity between the basal ganglia and the cerebellum (*t* = 3.1; df = 32; *p* = 0.004; *r* = 0.46) and between the basal ganglia and the somatosensory network (*t* = 3.0; df = 210; *p* = 0.003; *r* = 0.20). No significant connectivity alterations were found between the basal ganglia and the cortical motor network (*t* = 0.58; df = 313; *p* = 0.56; *r* = 0.03), the cerebellum and the somatosensory network (*t* = 2.3; df = 25; *p* = 0.028; *r* = 0.42), the cerebellum and the cortical motor network (*t* = 0.78; df = 33; *p* = 0.44; *r* = 0.13) or between the cortical motor and sensory network (*t* = 0.9; df = 213; *p* = 0.38; *r* = 0.06) (Figures 4, 5).

We further tested for changes between the thalamus and the sensory cortex, as well as between the thalamus and the cerebellar network. We found significantly increased connectivity between the thalamus and the somatosensory network (*t* = 3.6; df = 34; *p* < 0.001; *r* = 0.52), while the connectedness to the cerebellar network was decreased (*t* = 3.2; df = 30; *p* = 0.003; *r* = 0.50).

## Discussion

In the current study, we investigated the cerebral correlates of sensory–motor adaptation during an experimental intervention that induced a persistent and unresolveable sensory–motor mismatch (the subjects try to speak clearly but are unable to do so). The effects of a similar persistent mismatch after much longer time intervals (multiple days) were investigated in previous studies in patients who suffer from peripheral facial palsy. The following discussion focusses on similarities and differences between our short-term intervention and those long-term effects to delineate time-dependent effects and discuss the implications for our understanding of how the brain reacts to a persistent mismatch signal.

In line with the existing literature for long-term mismatch effects caused by facial palsy [after 14 days (6) and after 3–5 days (5)], we found increased connectedness within and between the striatal and cerebellar networks. The finding that the connectivity within and between these areas is altered after our short-term intervention fits well with our understanding of the roles that these areas play in motor learning. Cerebellar areas receive descending information from the cortical network during motor performance (efferent copy of planned movement), as well as ascending afferent information about the current sensory state. By integrating all this information, the cerebellum creates an internal predictive model of sensory states that guides learning by the trial-and-error principle (15, 16). This model allows predictive control by a feedforward mechanism in which the current sensory state and cortical motor commands are combined to forecast the future state of the body (17). The taping of the face in the current study prevents normal facial movements, and the cerebellar predictions become less precise. The increased prediction error is thought to be a major driver of motor learning, in which the striatum also plays a prominent role (18). It is not surprising that such a strong drive for motor learning is measurable shortly after the initiation of the mismatch, as these areas are key regions involved in the early phase of motor learning (18, 19). If the increased connectivity within the basal ganglia and cerebellum reflects an increased potential for motor learning, this could be useful for therapeutic applications. For example, a mismatch created by a virtual environment in which the movements of a hand deviate from real movements could cause a similar persistent mismatch that could be used as a therapeutic intervention.

However, in the current study, we also found differences between our results and previous results. The most striking difference from previous studies that investigated the effects of long-term mismatch was that we did not find clear evidence for the involvement of the cerebral motor cortex after this short period of time (neither within the motor cortex nor between the motor cortex and the striatum or the somatosensory cortex). Regarding the striato-cortical loop, we found reduced connectivity between the striatum and the somatosensory cortex. Concerning the cortico-cerebellar loop, we found a non-significant tendency for decreased connectivity. However, for both loops, we found no evidence for decreased connectedness to the motor cortex. The connectivity within the motor and somatosensory networks was not altered. In contrast, three previous studies in patients suffering from facial palsy found decreased connectedness in the somatosensory and motor network and between the motor network and the striatum after 14 days (6) and after 3–5 days (4, 5).

This result indicates that the short-term intervention that was used in the current study induced changes in the interpretation of somatosensory inputs but not in the motor program (or at least to a lesser degree). This finding is crucial because previous studies have demonstrated that successful error-based learning affects processing in both the motor and the somatosensory cortex in the very early stages of motor learning (18–20). The difference between successful and unsuccessful motor learning is that in successful error-based learning, a motor implementation that reduces the mismatch error is positively reinforced. In the case of an unsuccessful motor learning paradigm with persistent mismatch, there is no motor implementation that reduces the mismatch error, and accordingly, positive reinforcement is not able to change the current motor implementation. This might explain the lack of motor changes in the very early stages. However, the question of why the processing in the somatosensory cortex is altered remains. This question can be discussed in the context of the predictive coding view of information processing in the brain (21). The mismatch between the predicted sensory state after movement and the observed sensory state increases the prediction error. One of the main assumptions of our understanding of brain functioning is that the brain works to decrease this prediction error. Because we are not able to decrease the mismatch error by changing the motor program, we have to reduce the mismatch error by altering the desired somatosensory interpretation. However, measuring changes in the functional connectivity did not completely elucidate how this reduction is implemented. It is possible that the somatosensory interpretation of the current state is altered, but it is also possible that the decreased connectivity between the thalamus and somatosensory cortex indicates a reduced forwarding of the mismatch error.

Either way, the current results indicate that in the case of a persistent mismatch, the sensory interpretation of results is engaged first, while the motor implementation remains stable for a longer period of time. The cortical implementation and the mechanism of adaptation seem to be stepwise. However, this conclusion is only correct if the model we use here also represents and incorporates the major influencing variables of facial nerve palsy. An essential difference is a constant traction acting on the face in our experiment. This is not the case in a peripheral facial nerve palsy. Furthermore, the tape is not able to suppress all movements. This is similar to a peripheral facial nerve palsy, where slight movements are often possible. In our experiment, however, there is a clear relationship between the force used for facial movements and the range of motion achieved. This is not necessarily the case in peripheral facial nerve palsy. These deviations of our model from facial nerve palsy may also have contributed to different results in the motor system. To show the comparability of the effects of our model with peripheral facial nerve palsy for the motor system, and in particular to show the proposed gradual engagement of the somoatosensory and motor systems, a study of our experiment after a longer intervention period would be necessary.

A further point concerns the generalizability of our results. Given the current literature, we cannot be sure if this is a general mechanism or if it applies only to the face. Most studies on motor learning focus on limb movements, but the face is special. As an important example, muscle spindles were not found in facial muscles (22), and the role of proprioceptive feedback in facial learning is unclear. Mechanoreceptors that were described in the musculus zygomaticus and musculus buccalis in the human face might be at play here (23). As a second example, visual feedback and error signals resulting from visually observed failed limb movements are also lacking for facial processes because we rarely watch our own faces and even more rarely in spontaneous situations. How the face learns is, thus, an open and fascinating question. The current study provides a paradigm suitable for both healthy individuals and patients that might serve to answer this question in the future.

## Conclusion

**Figure 6:**
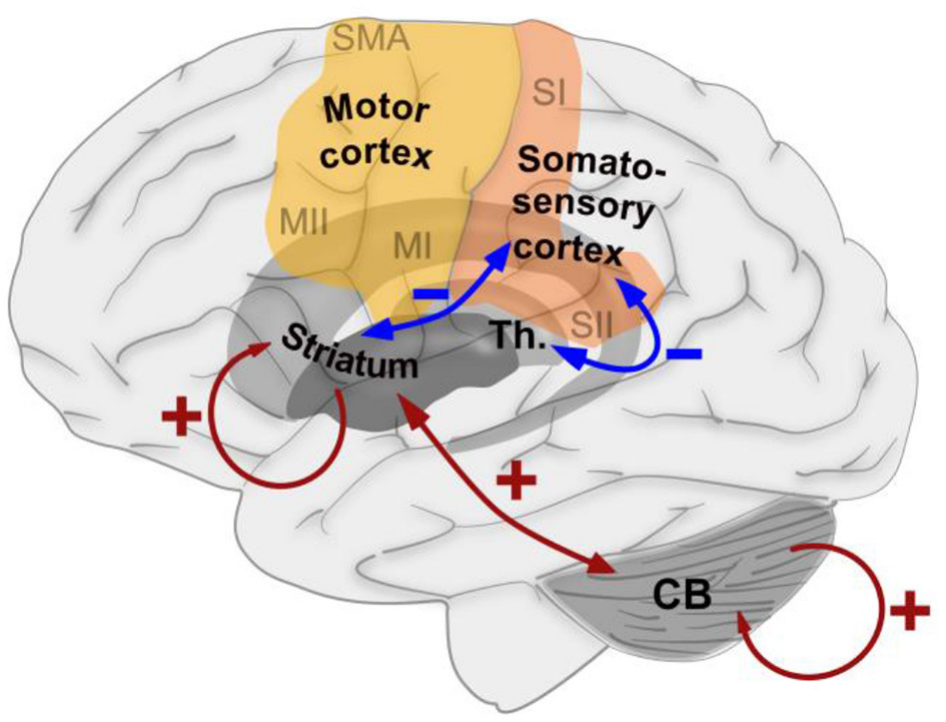
Schematic summary of the effects of exposure to a short-term, but persistent, sensory–motor mismatch on the functional connectedness between brain areas that were found in the current study. Blue arrows indicate decreased connectedness induced by the intervention, while red arrows indicate increased connectedness induced by the intervention.

In conclusion, we demonstrated that exposure to a short-term but persistent sensory–motor mismatch causes changes in cerebral information processing that are, to some extent, similar to those observed after days of persistent mismatch (Figure 6). The increased connectivity within the basal ganglia and cerebellum is suggestive of an increased potential for motor learning that might be useful for therapeutic application. Concerning the cortical sensory–motor connectivity, we found a decreased connectedness to the somatosensory cortex, while no significant changes were found in the motor cortex, suggesting a time-dependent mechanism in which the sensory interpretation of mismatch signals is affected first, while the motor implementation remains stable for a longer period of time.

## Data Availability Statement

The raw data supporting the conclusions of this article will be made available by the authors, without undue reservation.

## Ethics Statement

The studies involving human participants were reviewed and approved by Ethik-Kommission der Friedrich-Schiller-Universität Jena. The patients/participants provided their written informed consent to participate in this study.

## Author Contributions

CK, FK, LK, EJ, GV, SB, OG-L, OW, and CD contributed to the conception and design of the study. CK, FK, LK, and CD organized the data and conducted the experiments. CK and SB performed the data analysis. CK wrote the first draft of the manuscript. CK, EJ, GV, SB, OG-L, OW, and CD wrote sections of the manuscript. All authors contributed to manuscript revision, read, and approved the submitted version.

## Funding

OG-L acknowledges support by Deutsche Forschungsgemeinschaft (DFG) grant GU-463/12-1.

## Conflict of Interest

The authors declare that the research was conducted in the absence of any commercial or financial relationships that could be construed as a potential conflict of interest.

## Publisher's Note

All claims expressed in this article are solely those of the authors and do not necessarily represent those of their affiliated organizations, or those of the publisher, the editors and the reviewers. Any product that may be evaluated in this article, or claim that may be made by its manufacturer, is not guaranteed or endorsed by the publisher.
